# Aspirin or statin use in relation to survival after surgery for esophageal cancer: a population-based cohort study

**DOI:** 10.1186/s12885-023-10819-0

**Published:** 2023-04-25

**Authors:** Dag Holmberg, Eivind Gottlieb-Vedi, Jakob Hedberg, Mats Lindblad, Fredrik Mattsson, Jesper Lagergren

**Affiliations:** 1 Department of Molecular Medicine and Surgery, Karolinska Institutet and Karolinska University Hospital, Retzius Street 13A, 4Th Floor, 171 77 Stockholm, Sweden; 2 Department of Surgical Sciences, Uppsala University, Akademiska Sjukhuset, Uppsala, Sweden; 3 Department of Clinical Science, Intervention and Technology, Karolinska Institutet, Stockholm, Sweden; 4 Department of Upper Abdominal Diseases, Karolinska University Hospital, Stockholm, Sweden; 5 School of Cancer and Pharmacological Sciences, King’s College London, London, UK

**Keywords:** Esophageal neoplasm, Chemoprevention, Non-steroidal anti-inflammatory drugs, Chemotherapy, Adjuvant

## Abstract

**Background:**

Adjuvant postoperative treatment with aspirin and statins may improve survival in several solid tumors. This study aimed to assess whether these medications improve the survival after curatively intended treatment (including esophagectomy) for esophageal cancer in an unselected setting.

**Methods:**

This nationwide cohort study included nearly all patients who underwent esophagectomy for esophageal cancer in Sweden from 2006 to 2015, with complete follow-up throughout 2019. Risk of 5-year disease-specific mortality in users compared to non-users of aspirin and statins was analyzed using Cox regression, providing hazard ratios (HR) with 95% confidence intervals (CI). The HRs were adjusted for age, sex, education, calendar year, comorbidity, aspirin/statin use (mutual adjustment), tumor histology, pathological tumor stage, and neoadjuvant chemo(radio)therapy.

**Results:**

The cohort included 838 patients who survived at least 1 year after esophagectomy for esophageal cancer. Of these, 165 (19.7%) used aspirin and 187 (22.3%) used statins during the first postoperative year. Neither aspirin use (HR 0.92, 95% CI 0.67–1.28) nor statin use (HR 0.88, 95% CI 0.64–1.23) were associated with any statistically significant decreased 5-year disease-specific mortality. Analyses stratified by subgroups of age, sex, tumor stage, and tumor histology did not reveal any associations between aspirin or statin use and 5-year disease-specific mortality. Three years of preoperative use of aspirin (HR 1.26, 95% CI 0.98–1.65) or statins (HR 0.99, 95% CI 0.67–1.45) did not decrease the 5-year disease-specific mortality.

**Conclusions:**

Use of aspirin or statins might not improve the 5-year survival in surgically treated esophageal cancer patients.

**Supplementary Information:**

The online version contains supplementary material available at 10.1186/s12885-023-10819-0.

## Introduction

Esophageal cancer is common globally and carries a poor prognosis (< 20% 5-year survival), making it the sixth most common cause of cancer death in the world [1]. Surgical resection (esophagectomy), with or without neoadjuvant chemo(radio)therapy, offers the best chance of a cure for most of these patients. Nevertheless, the 5-year survival following such curatively intended treatment is only 30–45% [2, 3]. Regular and long-term medication with low-dose aspirin or statins, which are commonly used to prevent cardiovascular disease, has been associated with a reduced risk of developing several types of gastrointestinal cancer, including squamous cell carcinoma and adenocarcinoma of the esophagus [4–9]. Some research also indicates that post-treatment use of these medications may improve long-term survival. Therefore, aspirin and statins are being considered as adjuvant therapy to surgery for a range of solid neoplasias [10–15]. Yet, relatively few studies have assessed aspirin or statin medication following a diagnosis of esophageal cancer [16–20], and most of the available studies have had important methodological limitations, such as selection bias, confounding due to the inability to adjust for tumor stage and other factors, and time-related biases. Consequently, previous studies have provided widely conflicting results.

We aimed to test the hypothesis that aspirin and statin use is associated with decreased 5-year mortality in patients treated with esophagectomy for esophageal cancer in a study that took the main methodological concerns of the existing literature into account.

## Methods

### Design

This was a Swedish nationwide cohort study between July 1, 2006, and December 31, 2020. The cohort consisted of patients having undergone surgery for esophageal cancer. The exposures were the use of aspirin and statins, and the outcomes were disease-specific and all-cause mortality up to 5 years after surgery. Data were obtained from nationwide healthcare registries, which routinely and prospectively collect data on all healthcare in Sweden, as well as a review of medical records.

### Cohort

Potentially eligible patients were initially identified by the Swedish Cancer Registry and Swedish Patient Registry by the disease, histopathology, and surgery codes defining esophageal cancer (150 or 151.1 according to ICD-7) and esophagectomy (JCC00, JCC10, JCC11, JCC20, JCC30, JCC96, or JCC97 according to the NOMESCO Classification of Surgical Procedures). The study cohort was selected after a review of each patient’s medical records, including notes from histopathology reports, multidisciplinary meetings, surgery, and hospital discharge. The Swedish Cancer Registry provided tumor-specific data, including site, histopathology, stage, and date of diagnosis, and this registry has been validated with a 98% completeness for esophageal adenocarcinoma and esophageal squamous cell carcinoma [21]. The Swedish Patient Registry provided data on diagnoses and surgical procedures in in-patient and specialized out-patient healthcare, and this registry has been validated for generally high completeness and accuracy [22], and more specifically a positive predictive value for esophagectomy of 99.6% [23].

### Exposures

Aspirin and statin use were considered to be two separate exposures. Information about these medications was retrieved from the Swedish Prescribed Drug Registry, which automatically and electronically records all prescribed and dispensed medications in Swedish pharmacies with almost 100% completeness [24]. Aspirin use was defined by the dispensation of low-dose aspirin (75–160 mg daily) (Anatomical Therapeutic Chemical (ATC) code: B01AC06), and statin use was defined by the dispensation of a statin (ATC-codes: C10AA or C10BA). The exposures were assessed in different time periods with respect to the date of esophagectomy. The main exposure was any dispensation of aspirin or statin (henceforth “use”) during the first year following esophagectomy. The follow-up started one year after esophagectomy in order to avoid immortal time bias. We assessed potential duration-dependent associations by adding an analysis of long-term users, i.e., patients with aspirin or statin use at least one year preoperatively in addition to the first year after surgery. Finally, we examined the use of aspirin or statins for 1, 2, and 3 years prior to esophagectomy (independent of postoperative use) as secondary exposures. Low-dose aspirin and statins were only available by prescription in Sweden during the study period.

### Outcomes

The main outcome was 5-year disease-specific mortality, defined as death from esophageal cancer as an underlying or contributing cause of death within 5 years of the esophagectomy. We had data for disease-specific mortality until December 31, 2019. The secondary outcome was 5-year all-cause mortality, defined as any death occurring within 5 years of surgery. We preferred disease-specific mortality as the main outcome because the all-cause mortality might be biased by competing risk of mortality, particularly by a decreased risk of cardiovascular death in aspirin/statin users. The follow-up for all-cause mortality ended on December 31, 2020. Information on mortality was obtained from the Swedish Cause of Death Registry, which has 100% completeness for date of death and 96% completeness for cause of death, including deaths among Swedish residents who die abroad [25].

### Covariates

We considered the following nine covariates (with categorizations in parenthesis): age (continuous), sex (male or female), education (≤ 9, 10–12, or ≥ 13 years of formal education), calendar year (continuous), comorbidity (Charlson comorbidity index score 0, 1, or ≥ 2), aspirin or statin use (yes or no, mutual adjustment), tumor histology (adenocarcinoma or squamous cell carcinoma), pathological tumor stage (0-I, II, III, or IV), and neoadjuvant chemo(radio)therapy (yes or no). Data on age, sex, calendar year, and comorbidity were obtained from the Swedish Patient Registry. Education was assessed from the Swedish Longitudinal integrated database for health insurance and labour market studies (LISA) [26]. Comorbidity was classified based on the most well-validated version of the Charlson comorbidity index (Supplementary Table) [27]. Information on tumor histology, pathological tumor stage, and neoadjuvant chemo(radio)therapy were retrieved from a review of medical records.

### Statistical analysis

Follow-up started one year after the date of surgery and ended on the date of death, 5 years after surgery, or end of the study period, whichever occurred first. Cox proportional hazards models were used to calculate hazard ratios (HR) with 95% confidence intervals (CI), comparing the risk of mortality in users of aspirin or statins separately with non-users of these medications (reference groups). A multivariable model was adjusted for the nine covariates and categorizations presented above (Covariates). To further evaluate whether potential associations were modified by covariates, an interaction term was included in the models for the main exposure and each covariate where HRs were derived within each stratum. In these stratified analyses, HRs with 95% CI were derived within each stratum for age (≤ 59, 60–65, 66–71, and ≥ 72 years, with categories defined by quartiles, i.e., four similar-sized groups), sex (male or female), pathological tumor stage (0-I, II, III, and IV), and tumor histology (adenocarcinoma and squamous cell carcinoma). This was done for each covariate separately. Because missing data were low (found in at least one covariate in only 2% of patients), we conducted a complete case analysis, i.e., excluded patients with missing data in any variable. The proportional hazards assumption was evaluated using log–log survival plots and by calculating the correlations between Schoenfeld residuals for covariates and ranking of individual failure time. The low correlations showed that the proportional hazards assumption was met for all analyses. A senior biostatistician (FM) conducted the data management and statistical analyses according to a detailed and pre-defined study protocol and used the statistical software SAS/STAT Statistical Package, Version 9.4 (SAS Institute Inc., Cary, NC, USA).

## Results

### Patients

**Fig. 1:**
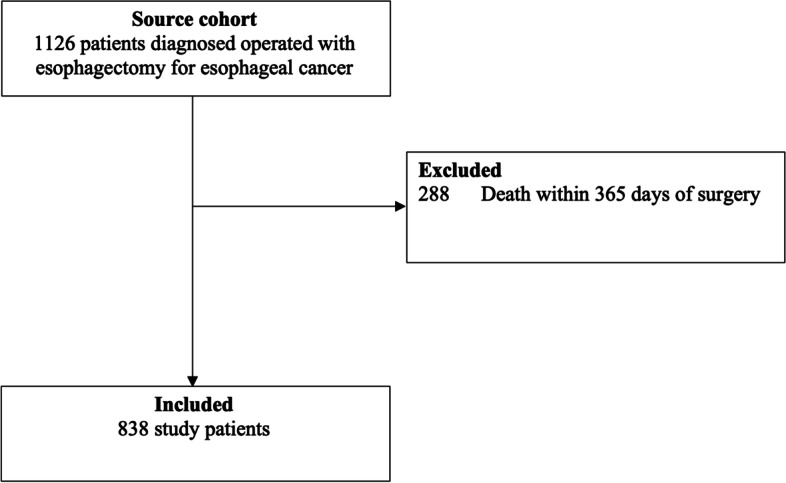
Study population flowchart

Among 1,126 patients who underwent esophagectomy (with or without chemo(radio)therapy) for esophageal cancer, 838 (74.4%) patients survived the first year after surgery and thus remained for final analysis (Fig. 1). In total, 165 (19.7%) patients used aspirin, 187 (22.3%) used statins, and 70 (8.4%) used both aspirin and statins during the first postoperative year. The mean age was 64.8 years (standard deviation 9.2), a majority of patients (80.0%) were men, and the overall 5-year survival was 38.7%. Compared to non-users, both aspirin users and statin users had higher frequencies of men, comorbidity, use of the other medication (aspirin or statins), squamous cell carcinoma histology, and non-users of neoadjuvant chemo(radio)therapy (Table 1).

**Table 1:** Characteristics of 838 patients who survived at least one year after esophagectomy for esophageal cancer, stratified by use of aspirin and statins during the first postoperative year

	**Number (%)**		**Number (%)**	
	**Non-users of aspirin**	**Aspirin users**	**Non-users of statin**	**Statin users**
**Total**	673 (100.0)	165 (100.0)	651 (100.0)	187 (100.0)
**Total years of follow-up**	1829 (100.0)	458 (100.0)	1758 (100.0)	530 (100.0)
**Mean age (standard deviation)**	64.0 (9.5)	67.9 (7.1)	63.9 (9.4)	67.7 (7.8)
**Sex**				
Men	509 (75.6)	148 (89.7)	492 (75.6)	165 (88.2)
Women	164 (24.4)	17 (10.3)	159 (24.4)	22 (11.8)
**Years of education**				
≤ 9	223 (33.1)	62 (37.6)	212 (32.6)	73 (39.0)
10–12	298 (44.3)	72 (43.6)	292 (44.8)	78 (41.7)
≥ 13	149 (22.1)	31 (18.8)	144 (22.1)	36 (19.3)
Missing	3 (0.5)	0 (0.0)	3 (0.5)	0 (0.0)
**Calendar period**				
< 2012	283 (42.1)	80 (48.5)	265 (40.7)	98 (52.4)
≥ 2012	390 (57.9)	85 (51.5)	386 (59.3)	89 (47.6)
**Charlson comorbidity index**				
0	332 (49.3)	32 (19.4)	317 (48.7)	47 (25.1)
1	219 (32.5)	59 (35.8)	215 (33.0)	63 (33.7)
≥ 2	122 (18.1)	74 (44.9)	119 (18.3)	77 (41.2)
**Aspirin/statin use**				
Yes	84 (12.5)	103 (62.4)	62 (9.5)	103 (55.1)
No	589 (87.5)	62 (37.6)	589 (90.5)	84 (44.9)
**Pathological tumor stage**				
0-I	280 (41.6)	66 (40.0)	270 (41.5)	76 (40.6)
II	143 (21.3)	40 (24.2)	141 (21.6)	42 (22.5)
III	183 (27.2)	46 (27.9)	171 (26.3)	58 (31.0)
IV	58 (8.6)	12 (7.3)	60 (9.2)	10 (5.4)
Missing	9 (1.3)	1 (0.6)	9 (1.4)	1 (0.5)
**Tumor histology**				
Adenocarcinoma	493 (73.3)	130 (78.8)	469 (72.0)	154 (82.4)
Squamous cell carcinoma	176 (26.2)	35 (21.2)	178 (27.4)	33 (17.6)
Missing	4 (0.6)	0 (0.0)	4 (0.6)	0 (0.0)
**Neoadjuvant chemo(radio)therapy**				
Yes	236 (34.1)	90 (54.6)	433 (66.5)	94 (50.3)
No	437 (64.9)	75 (45.4)	218 (33.5)	93 (49.7)

### Aspirin use and 5-year mortality

**Fig. 2:**
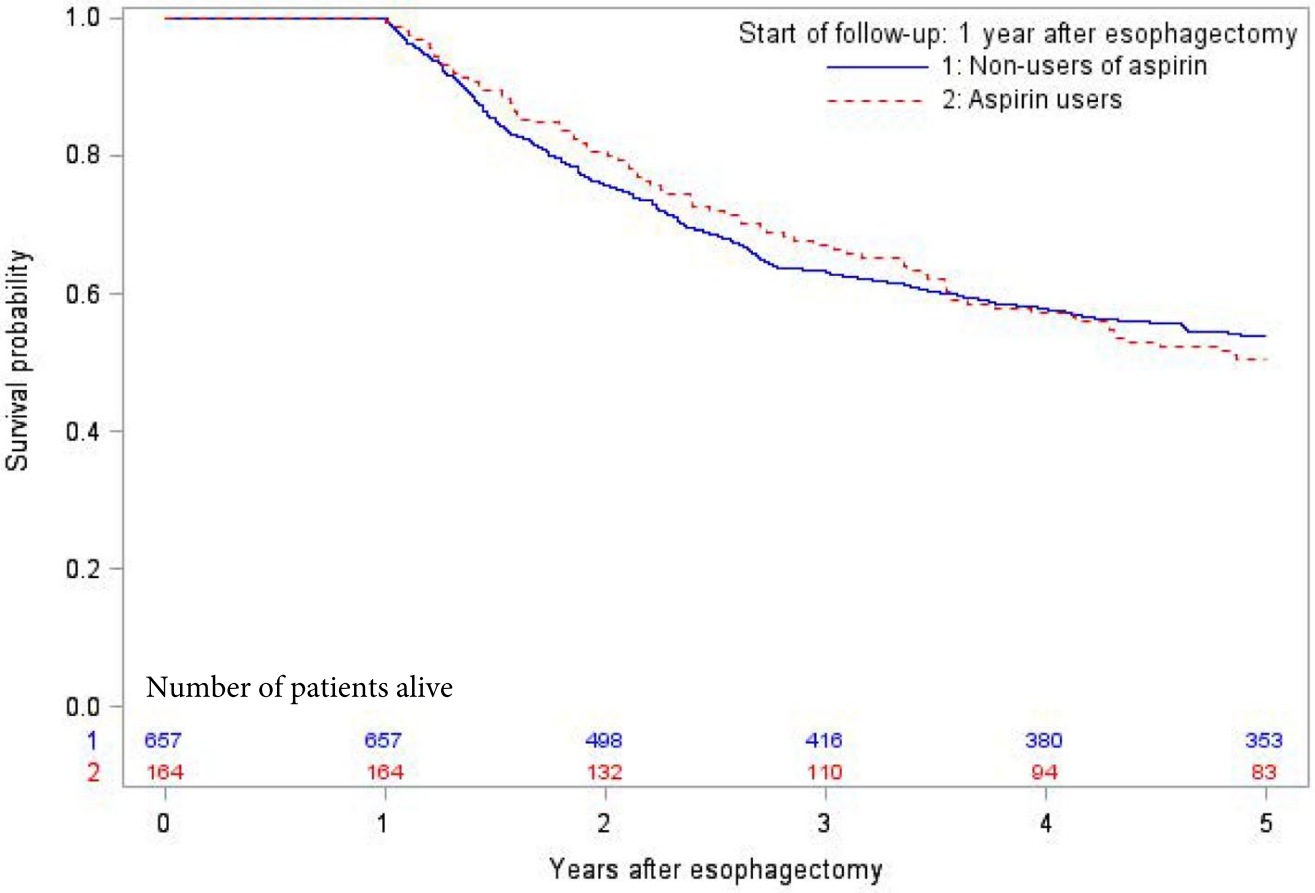
Kaplan–Meier survival estimates of disease-specific survival among 1-year survivors in surgically treated esophageal cancer as a function of time, stratified by postoperative aspirin use

The cumulative 5-year survival probability as a function of time for aspirin and non-aspirin users is presented in Fig. 2. Aspirin use for one year after surgery for esophageal cancer was not associated with any statistically significantly decreased risk of 5-year disease-specific mortality (adjusted HR 0.92, 95% CI 0.67–1.28) or 5-year all-cause mortality (adjusted HR 0.92, 95% CI 0.69–1.21) (Table 2). Stratified analyses did not show any decreased risk of 5-year disease-specific mortality in subgroups of age, sex, tumor stage, or tumor histology (Table 2). Patients using aspirin both during the year before and the year after surgery had similar 5-year disease-specific mortality compared to patients not using aspirin during the same time period (adjusted HR 1.02, 95% CI 0.71–1.46). Aspirin use for 1, 2, or 3 years before surgery was not associated with any decreased adjusted HR of 5-year disease-specific mortality (HR 1.26, 95% CI 0.92–1.74 for 1 year; HR 1.30, 95% CI 1.02–1.66 for 2 years; and HR 1.26, 95% CI 0.98–1.65 for 3 years before surgery) compared to non-use during the corresponding time periods.

**Table 2:** Aspirin use and risk of 5-year disease-specific mortality after esophagectomy for esophageal cancer (complete case analysis)

	**Number**		**Hazard ratio (95% confidence interval)**	
	**At risk**	**Deaths**	**Unadjusted**	**Adjusted**^**a**^
**All patients**				
No aspirin	657	225	1.00 (Reference)	1.00 (Reference)
Aspirin	164	62	1.07 (0.81–1.42)	0.92 (0.67–1.28)
**Age ≤ 59 years**				
No aspirin	197	56	1.00 (Reference)	1.00 (Reference)
Aspirin	23	8	0.95 (0.47–1.93)	1.04 (0.47–2.29)
**Age 60–65 years**				
No aspirin	154	57	1.00 (Reference)	1.00 (Reference)
Aspirin	38	18	1.50 (0.93–2.43)	1.28 (0.72–2.27)
**Age 66–71 years**				
No aspirin	168	59	1.00 (Reference)	1.00 (Reference)
Aspirin	61	20	0.88 (0.56–1.39)	0.78 (0.46–1.34)
**Age ≥ 72 years**				
No aspirin	138	53	1.00 (Reference)	1.00 (Reference)
Aspirin	42	16	1.06 (0.64–1.77)	0.90 (0.50–1.63)
**Men**				
No aspirin	498	183	1.00 (Reference)	1.00 (Reference)
Aspirin	147	59	1.15 (0.86–1.53)	0.99 (0.70–1.40)
**Women**				
No aspirin	159	42	1.00 (Reference)	1.00 (Reference)
Aspirin	17	3	0.44 (0.14–1.37)	0.62 (0.19–2.02)
**Tumor stage 0-I**				
No aspirin	275	58	1.00 (Reference)	1.00 (Reference)
Aspirin	66	12	0.44 (0.25–0.79)	0.78 (0.41–1.48)
**Tumor stage II**				
No aspirin	142	45	1.00 (Reference)	1.00 (Reference)
Aspirin	40	18	1.28 (0.79–2.07)	1.30 (0.73–2.33)
**Tumor stage III**				
No aspirin	182	91	1.00 (Reference)	1.00 (Reference)
Aspirin	46	25	1.92 (1.27–2.90)	0.98 (0.60–1.61)
**Tumor stage IV**				
No aspirin	58	31	1.00 (Reference)	1.00 (Reference)
Aspirin	12	7	1.77 (0.83–3.75)	0.70 (0.30–1.62)
**Adenocarcinoma histology**				
No aspirin	488	160	1.00 (Reference)	1.00 (Reference)
Aspirin	129	45	0.97 (0.70–1.33)	0.98 (0.67–1.144)
**Squamous cell carcinoma histology**				
No aspirin	169	65	1.00 (Reference)	1.00 (Reference)
Aspirin	35	17	1.47 (0.90–2.40)	0.88 (0.50–1.57)

^a^Adjusted for age, sex, education level, calendar year, comorbidity, statin use, tumor histology, tumor stage, and neoadjuvant chemoradiotherapy

### Statin use and 5-year mortality

**Fig. 3:**
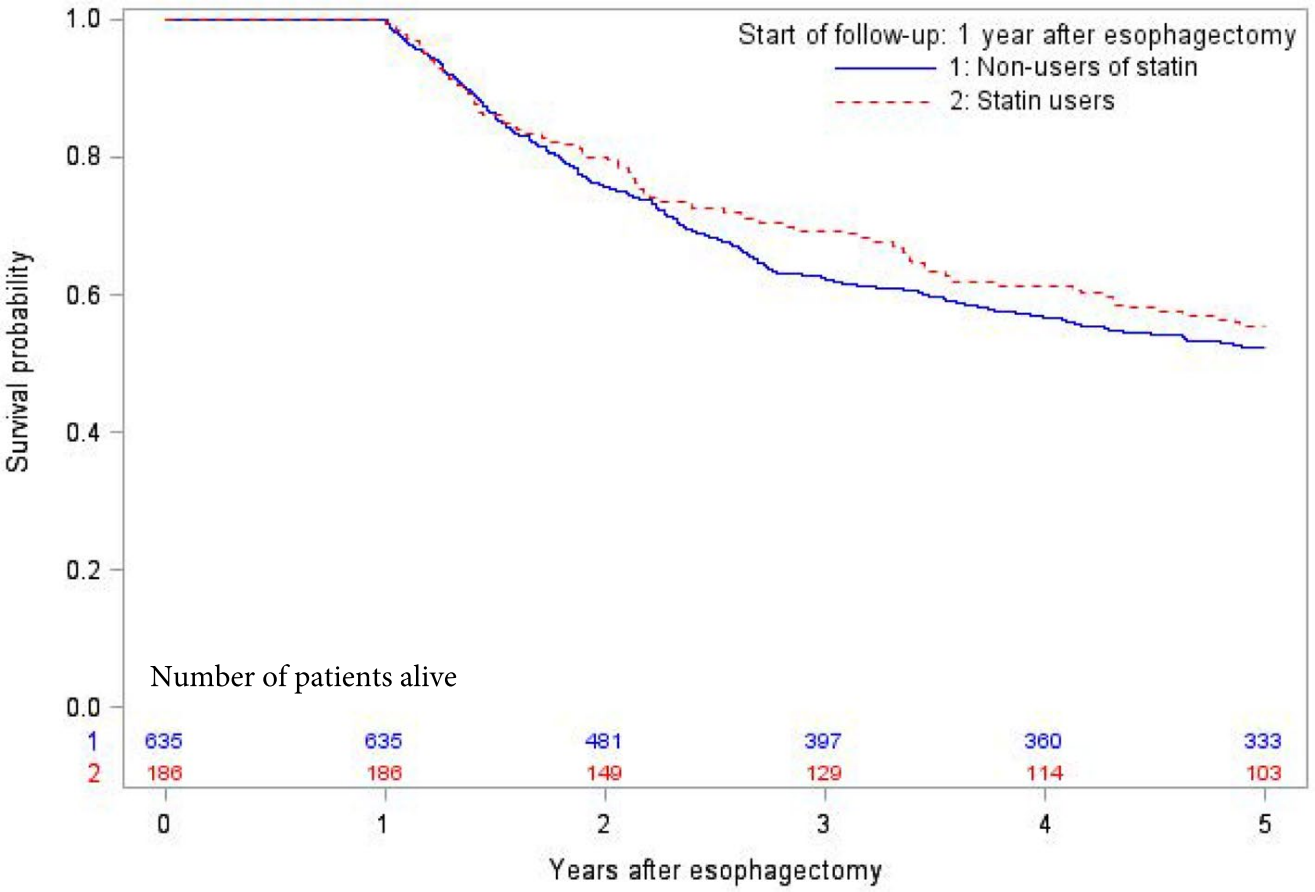
Kaplan–Meier survival estimates of disease-specific survival among 1-year survivors in surgically treated esophageal cancer as a function of time, stratified by postoperative statin use

The cumulative 5-year survival probability as a function of time for statin and non-statin users is presented in Fig. 3. Statin use one year after surgery was not associated with any statistically significantly decreased risk of 5-year disease-specific mortality (adjusted HR 0.88, 95% CI 0.64–1.23) or 5-year all-cause mortality (adjusted HR 0.81, 95% CI 0.61–1.09) (Table 3). Stratified analyses did not reveal any decreased risk of 5-year disease-specific mortality in any subgroup of age, sex, tumor stage, or tumor histology (Table 3). Patients using statins both during the year before and the year after surgery had similar 5-year disease-specific survival compared to patients not using statins during this time period (adjusted HR 0.89, 95% CI 0.62–1.28). Statin use for 1, 2, or 3 years before surgery was not associated with any decreased adjusted HR of 5-year disease-specific mortality (HR 0.95, 95% CI 0.70–1.28 for 1 year; HR 1.06, 95% CI 0.76–1.48 for 2 years; and HR 0.99, 95% CI 0.67–1.45 for 3 years before surgery) compared to non-use of statins during the corresponding time periods.

**Table 3:** Statin use and risk of 5-year disease-specific mortality after esophagectomy for esophageal cancer (complete case analysis)

	**Number**		**Hazard ratio (95% confidence interval)**	
	**At risk**	**Deaths**	**Unadjusted**	**Adjusted**^**a**^
**All patients**				
No statins	635	221	1.00 (Reference)	1.00 (Reference)
Statins	186	66	0.98 (0.75–1.30)	0.88 (0.64–1.23)
**Age ≤ 59 years**				
No statins	189	58	1.00 (Reference)	1.00 (Reference)
Statins	31	6	0.47 (0.21–1.05)	0.48 (0.20–1.13)
**Age 60–65 years**				
No statins	154	61	1.00 (Reference)	1.00 (Reference)
Statins	38	14	1.07 (0.62–1.84)	0.90 (0.48–1.67)
**Age 66–71 years**				
No statins	164	56	1.00 (Reference)	1.00 (Reference)
Statins	65	23	0.98 (0.64–1.51)	0.96 (0.57–1.61)
**Age ≥ 72 years**				
No statins	128	46	1.00 (Reference)	1.00 (Reference)
Statins	52	23	1.29 (0.84–1.98)	1.09 (0.64–1.87)
**Men**				
No statins	481	183	1.00 (Reference)	1.00 (Reference)
Statins	164	59	0.99 (0.75–1.33)	0.86 (0.61–1.22)
**Women**				
No statins	154	38	1.00 (Reference)	1.00 (Reference)
Statins	22	7	0.87 (0.41–1.85)	1.08 (0.47–2.48)
**Tumor stage 0-I**				
No statins	265	57	1.00 (Reference)	1.00 (Reference)
Statins	76	13	0.41 (0.23–0.71)	0.70 (0.38–1.31)
**Tumor stage II**				
No statins	140	43	1.00 (Reference)	1.00 (Reference)
Statins	42	20	1.34 (0.85–2.12)	1.51 (0.85–2.66)
**Tumor stage III**				
No statins	170	87	1.00 (Reference)	1.00 (Reference)
Statins	58	29	1.66 (1.13–0.44)	0.80 (0.51–1.28)
**Tumor stage IV**				
No statins	60	34	1.00 (Reference)	1.00 (Reference)
Statins	10	4	1.42 (0.53–3.82)	0.61 (0.21–1.78)
**Adenocarcinoma**				
No statins	464	153	1.00 (Reference)	1.00 (Reference)
Statins	153	52	0.93 (0.69–1.26)	0.94 (0.66–1.35)
**Squamous cell carcinoma**				
No statins	171	68	1.00 (Reference)	1.00 (Reference)
Statins	33	14	1.24 (0.72–2.12)	0.72 (0.39–1.34)

^a^ Adjusted for age, sex, education level, calendar year, comorbidity, aspirin use, tumor histology, tumor stage, and neoadjuvant chemoradiotherapy

## Discussion

This study showed no 5-year survival benefit from one-year use of aspirin or statins after esophagectomy for esophageal cancer. This finding was corroborated in subgroups of age, sex, tumor stage, tumor histology, and analyses of preoperative users of aspirin or statins.

Main methodological advantages of this study compared to existing studies were the population-based design which counteracted selection bias, the adjustment for all major known prognostic factors (including tumor stage), and the measures taken to avoid time bias. By combining information from well-maintained national complete registries with that of medical records, data on exposures, outcomes, and covariates were detailed and of high quality, thus minimizing misclassification. The similar results in the subgroup analyses and when examining post- and pre-operative and long-term use of aspirin and statins show a consistency of the negative findings. Given the nationwide coverage and the unselected patient sample with a low proportion of missing data, the results may be generalizable to countries with similar demographics and healthcare as in Sweden, i.e., Northern Europe. Among weaknesses is the risk of confounding by indication. Aspirin and statins are typically prescribed to patients at risk of cardio- and cerebrovascular diseases and may therefore be associated with poorer survival due to such diseases. However, this risk was reduced by the adjustment of comorbidity and other covariates, and by assessing disease-specific mortality as the main outcome. There was also a risk of exposure misclassification because high-dose aspirin and other non-steroid anti-inflammatory drugs (but not low-dose aspirin) are available over the counter which might wrongly drive the present HRs toward the null. However, high-dose aspirin and non-steroid anti-inflammatory drugs are usually taken on demand for shorter periods, which should limit any impact on the 5-year mortality outcomes. Finally, we cannot exclude minor protective effects of aspirin or statins due to limited statistical power. However, the point estimates were close to unity and the results were consistent across sub-analyses, indicating robustness.

Aspirin exerts its mechanism of action by irreversibly inhibiting the cyclooxygenase (COX) 1 and 2 enzymes, which are regularly upregulated in esophageal cancer [28]. Inhibition of these enzymes leads to decreased platelet aggregation and decreased levels of circulating, pro-inflammatory prostaglandins, both of which have been proposed to play important roles in tumor biology. Only a few observational studies have investigated if the use of aspirin or statins before or after a diagnosis of esophageal cancer improves survival, albeit with conflicting results. Regarding aspirin, a Dutch study of 946 esophageal cancer patients found that post-diagnosis aspirin use, defined as a time-varying covariate, was associated with a strongly decreased risk of mortality in esophageal adenocarcinoma (HR 0.24, 95% CI 0.10–0.59), but not in esophageal squamous cell carcinoma (HR 1.02, 95% CI 0.37–2.83) [16]. However, a decreased mortality of 76% in adenocarcinoma seems unrealistic. Use of time-varying exposures may have resulted in reverse causation, e.g., patients may contribute exposed person-time on aspirin or statins after surgery only until tumors recur. The use of aspirin or statins is often discontinued if the tumors recur and the patients deteriorate, and thus, these patients contribute the remaining short time of life with unexposed person-time and the death becomes wrongly attributed to non-use of medications. Thus, the use of time-varying definitions of aspirin and statins after a cancer diagnosis may not be appropriate. In similarity to the present study, a large British cohort study that undertook steps to avoid time bias found no association between aspirin use and mortality in 4,654 surgically treated esophageal cancer patients [17]. However, data on tumor stage, which is by far the most powerful predictor of survival, were missing in 80% of patients in that study, which made the results susceptible to confounding.

Statins reduce serum cholesterol through inhibition of the mevalonate pathway, which could lead to decreased cancer cell proliferation and migration and reduction in the risk of metastasis and cancer mortality [29]. A population-based study in the United Kingdom of 1,165 patients with esophageal cancer showed that statin use was associated with a 38% decreased risk of disease-specific mortality in patients with adenocarcinoma (HR 0.62, 95% CI 0.44–0.86), while no association with squamous cell carcinoma was found [18]. That study did not adjust for tumor stage and was prone to bias due to reverse causation. A Scottish cohort study of 1,921 esophageal cancer patients that accounted for reverse causation found no association between post-diagnosis use of statins and mortality. However, that study also lacked adjustment for tumor stage [19]. A large Belgian cohort study of 5,234 patients with esophageal cancer that controlled for immortal time bias and tumor stage found statin use to be associated with a slightly decreased risk of disease-specific mortality (HR 0.87, 95% CI 0.78–0.97) [20]. Combining the findings of the present study with the previous literature makes it reasonable to argue that any prognostic benefit of aspirin or statins is negligible.

## Conclusion

In conclusion, the findings from this population-based cohort study, with measures taken to avoid selection bias, confounding by the main prognostic factors, and time bias do not support the hypothesis of a 5-year survival benefit of using low-dose aspirin or statins following esophagectomy for esophageal cancer.

## Supplementary Information

**Additional file 1.**

## Data Availability

Individual level data are not available from the authors. Registry data are available from the National Board of Health & Welfare (https://www.socialstyrelsen.se/en/).

## Abbreviations

ATC: Anatomical Therapeutic Chemical
CI: Confidence interval
HR: Hazard ratio
LISA: Longitudinal integrated database for health insurance and labour market studies

## References

[1] Lagergren J, Smyth E, Cunningham D (2017). Oesophageal cancer. Lancet.

[2] Coleman HG, Xie SH, Lagergren J (2018). The Epidemiology of Esophageal Adenocarcinoma. Gastroenterology.

[3] Lagergren J (2015). Oesophageal cancer in 2014: Advances in curatively intended treatment. Nat Rev Gastroenterol Hepatol.

[4] Algra AM, Rothwell PM (2012). Effects of regular aspirin on long-term cancer incidence and metastasis: a systematic comparison of evidence from observational studies versus randomised trials. Lancet Oncol.

[5] Corley DA, Kerlikowske K, Verma R (2003). Protective association of aspirin/NSAIDs and esophageal cancer: a systematic review and meta-analysis. Gastroenterology.

[6] Liao LM, Vaughan TL, Corley DA, et al. Nonsteroidal anti-inflammatory drug use reduces risk of adenocarcinomas of the esophagus and esophagogastric junction in a pooled analysis. Gastroenterology. 2012;142:442–452 e5; quiz e22–3. 10.1053/j.gastro.2011.11.019 PMC3488768 22108196

[7] Alexandre L, Clark AB, Bhutta HY (2014). Statin use is associated with reduced risk of histologic subtypes of esophageal cancer: a nested case-control analysis. Gastroenterology.

[8] Hippisley-Cox J, Coupland C (2010). Unintended effects of statins in men and women in England and Wales: population based cohort study using the QResearch database. BMJ.

[9] Singh S, Singh AG, Singh PP, et al. Statins are associated with reduced risk of esophageal cancer, particularly in patients with Barrett’s esophagus: a systematic review and meta-analysis. Clin Gastroenterol Hepatol. 2013;11:620–9. 10.1016/j.cgh.2012.12.036 PMC3660516 23357487

[10] Hua X, Phipps AI, Burnett-Hartman AN (2017). Timing of Aspirin and Other Nonsteroidal Anti-Inflammatory Drug Use Among Patients With Colorectal Cancer in Relation to Tumor Markers and Survival. J Clin Oncol.

[11] Coyle C, Cafferty FH, Rowley S (2016). ADD-ASPIRIN: A phase III, double-blind, placebo controlled, randomised trial assessing the effects of aspirin on disease recurrence and survival after primary therapy in common non-metastatic solid tumours. Contemp Clin Trials.

[12] Liao X, Lochhead P, Nishihara R (2012). Aspirin use, tumor PIK3CA mutation, and colorectal-cancer survival. N Engl J Med.

[13] Alexandre L, Clark AB, Walton S (2020). Adjuvant statin therapy for oesophageal adenocarcinoma: the STAT-ROC feasibility study. BJS Open.

[14] Kim ST, Kang JH, Lee J (2014). Simvastatin plus capecitabine-cisplatin versus placebo plus capecitabine-cisplatin in patients with previously untreated advanced gastric cancer: a double-blind randomised phase 3 study. Eur J Cancer.

[15] Lim SH, Kim TW, Hong YS (2015). A randomised, double-blind, placebo-controlled multi-centre phase III trial of XELIRI/FOLFIRI plus simvastatin for patients with metastatic colorectal cancer. Br J Cancer.

[16] Frouws MA, Bastiaannet E, Langley RE (2017). Effect of low-dose aspirin use on survival of patients with gastrointestinal malignancies; an observational study. Br J Cancer.

[17] Spence AD, Busby J, Johnston BT (2018). Low-Dose Aspirin Use Does Not Increase Survival in 2 Independent Population-Based Cohorts of Patients With Esophageal or Gastric Cancer. Gastroenterology.

[18] Alexandre L, Clark AB, Bhutta HY, et al. Association Between Statin Use After Diagnosis of Esophageal Cancer and Survival: A Population-Based Cohort Study. Gastroenterology. 2016;150:854–65 e1; quiz e16–7. 10.1053/j.gastro.2015.12.039 26775632

[19] Cardwell CR, Spence AD, Hughes CM (2017). Statin use after esophageal cancer diagnosis and survival: A population based cohort study. Cancer Epidemiol.

[20] Lacroix O, Couttenier A, Vaes E (2019). Statin use after diagnosis is associated with an increased survival in esophageal cancer patients: a Belgian population-based study. Cancer Causes Control.

[21] Lindblad M, Ye WM, Lindgren AS (2006). Disparities in the classification of esophageal and cardia adenocarcinomas and their influence on reported incidence rates. Ann Surg.

[22] Ludvigsson JF, Andersson E, Ekbom A (2011). External review and validation of the Swedish national inpatient register. BMC Public Health.

[23] Lagergren K, Derogar M (2012). Validation of oesophageal cancer surgery data in the Swedish Patient Registry. Acta Oncol.

[24] Wettermark B, Hammar N, Fored CM (2007). The new Swedish Prescribed Drug Register–opportunities for pharmacoepidemiological research and experience from the first six months. Pharmacoepidemiol Drug Saf.

[25] Brooke HL, Talback M, Hornblad J (2017). The Swedish cause of death register. Eur J Epidemiol.

[26] Ludvigsson JF, Svedberg P, Olén O (2019). The longitudinal integrated database for health insurance and labour market studies (LISA) and its use in medical research. Eur J Epidemiol.

[27] Brusselaers N, Lagergren J (2017). The Charlson Comorbidity Index in Registry-based Research. Methods Inf Med.

[28] van Staalduinen J, Frouws M, Reimers M (2016). The effect of aspirin and nonsteroidal anti-inflammatory drug use after diagnosis on survival of oesophageal cancer patients. Br J Cancer.

[29] Nielsen SF, Nordestgaard BG, Bojesen SE (2012). Statin Use and Reduced Cancer-Related Mortality. N Engl J Med.

