# Supplementary material for: Aspirin or statin use in relation to survival after surgery for esophageal cancer: a population-based cohort study
Source: BMC Cancer. 2023 Apr 25;23:375. doi: 10.1186/s12885-023-10819-0 (PMC10127395; doi:10.1186/s12885-023-10819-0)
Supplement: Supplementary file 1 — Additional file 1. [file 12885_2023_10819_MOESM1_ESM.docx]

| **Supplementary Table. Diagnoses and corresponding ICD-codes for Charlson comorbidity index in Sweden.** | |
| --- | --- |
|  | **ICD-10** |
| **Years of use** | 1997-present |
|  |  |
| **Myocardial Infarction** | I21-I23, I252 |
| **Congestive Heart Failure** | I11, I13, I255, I42-I43, I50, I517 |
| **Peripheral Vascular Disease** | I70–I73, I770–I771, K551, K558– K559, R02, Z958–Z959 |
| **Cerebrovascular Disease** | G45–G46, I60–I69 |
| **Dementia** | A810, F00–F03, F051, G30–G31 |
| **Chronic Pulmonary Disease** | I26–I27, J40–J47, J60–J67, J684, J701, J703 |
| **Rheumatic Disease** | M05–M06, M09, M120, M315, M32–M36 |
| **Liver Disease** | B18, I85, I864, I982, K70–K71, K721, K729, K76, R162, Z944 |
| **Hemiplegia** | G114, G81–G83 |
| **Renal Disease** | I12–I13, N01, N03, N05, N07–N08, N171–N172, N18, N19, N25, Z49, Z940, Z992 |
| **Any Malignancy** | C00–C26, C30–C34, C37–C41, C43, C45–C58, C60–C76, C80–C85, C88, C90–C97 |
| **Metastatic Tumors** | C77–C79 |
| **AIDS** | B20–B24 |
| Note: The search for ICD codes was limited to three years prior to study entry. | |
